# Physical and psychological status of emergency assistance personnel at major public health events: a qualitative descriptive study

**DOI:** 10.1186/s12889-024-19367-z

**Published:** 2024-07-18

**Authors:** Chen Qiu, Linyue Zhang, Peiyao Qi, Yu Miao, Hao Han, Xingxing Hu, Yuan Gao, Xuemei Li

**Affiliations:** 1 Department of Orthopedics, The Fourth Medical Center, Chinese PLA General Hospital, Beijing, China; 2 Department of Cardiology, The Second Medical Center, Chinese PLA General Hospital, Beijing, China; 3 Department of Endocrinology, The Second Medical Center, Chinese PLA General Hospital, Beijing, China; 4 Unit 65316 of Chinese PLA, Dalian, China; 5 Unit 66325 of Chinese PLA, Beijing, China; 6 Department of Nursing, The First Medical Center, Chinese PLA General Hospital, No.28 Fuxing Road, Haidian District, Beijing, 100853 China; 7 Faculty of Nursing, Naval Medical University, No.800 Xiangyin Road, Yangpu District, Shanghai, 200433 China

**Keywords:** Public health, Rescue work, Psychological interview, Qualitative research

## Abstract

**Background:**

Many personnel respond to natural disasters like earthquakes and tsunamis and frequent public health events like Ebola and COVID-19. However, research on emergency assistance personnel remains limited. This study aims to describe the perceived well-being among responders deployed in isolated or emergency international missions while providing practical references to intervene in developing similar missions.

**Methods:**

For this qualitative phenomenological study, purposive sampling was used following the principle of maximum differentiation to select personnel deployed on an emergency mission for over a year. Data collection continued until data saturation. Phenomenologically semi-structured interviews helped explore the physical and psychological status of the participants with Colaizzi’s method.

**Results:**

Eleven personnel were interviewed after the mission, with four major themes being identified: ‘perceived somatic change,’ ‘perceived emotional change,’ ‘behavioral change,’ and ‘coping with perceived change.’

**Conclusions:**

The mental health status of the emergency assistance personnel was affected by multiple factors from external and internal environments. The current study explored the physical and psychological feelings and emotions of emergency assistance personnel during an emergency mission. The study provided a practical reference for health management under similar missions.

**Registrations:**

Not registered.

## Background

Many personnel are required for various emergency security or rescue missions to respond to natural disasters such as earthquakes and tsunamis and frequent public health events such as Ebola and COVID-19. The psychological problems of such emergency assistance personnel require urgent attention from researchers due to the specificity of emergency security and rescue missions regarding time, space, and content. This is because emergencies usually occur suddenly. Thus, most emergency assistance personnel are immediately deployed, while professional training or adequate psychological preparation requires more time. Emergency assistance personnel often operate far from their familiar working and living environment. All of these objective conditions need personnel to face the challenge of adapting quickly to different climates and cultures. Moreover, the personnel deployed are on their first mission and thus need to gain relevant experience to draw on. Their uneasiness will increase because of the risk uncertainty of the task. In light of the specificity of the tasks and the need for security, the personnel are managed separately and often in an environment isolated from the outside, which adds to their psychological burden.

In particular, emergency assistance for major public health events is similar to military missions, with similar psychological triggers. Prior studies have reported that soldiers involved in special military missions such as disaster rescue, maneuvers, and joint exercises are at high risk of mental health problems. The strict top-down command system of military organizations, poor service environment, sustained operations, and job inappropriateness make it challenging for soldiers to adapt to the forces, evoking possible stress reactions. This can lead to psychological problems such as emotional disorders, depression, post-traumatic stress disorder, and substance and alcohol abuse [1, 2], the leading causes of suicide and attrition among military personnel. These problems could directly affect the task execution efficiency [3]. The phenomenon is more noticeable in countries such as South Korea, where enlistment is mandatory rather than voluntary [4]. During World War I, military psychology developed as a specialty in the UK and the US, describing the application of psychological science to military operations, systems, and personnel [5, 6]. Mental health is a critical component of military medical protection worldwide. In 2006, the Ministry of National Defense of China formally included psychological screening in its recruitment tests.

A summary of previous studies reveals that emergency responders are at a greater risk of poor physical and mental health, decreased cognitive function, and elevated injuries [7, 8]. The precipitating factors of psychological problems can be categorized into internal and external factors. Prolonged physical activity, deficient energy intake combined with inadequate sleep can result in an impaired emotional state [9]. In addition, the effects of external factors such as living environment, mission pressure, and social support cannot be ignored [1, 10]. Global aid organizations effectively evaluate, identify, and prevent psychological problems. Currently, scales such as the Symptom Check list-90, Post-Traumatic Stress Disorder Checklist-Civilian Version, the Self-Rating Depression Scale, and the Self-Rating Anxiety Scale are used to complete psychological assessments [11–13]. However, these scales are universal and are not pertinent to the specific needs of soldier assessment. Chinese researchers [3] have constructed an early warning indicator for psychological crises in soldiers, which facilitates targeting individuals prone to psychological problems. Reducing the safety risks induced by psychological problems and ensuring mission completion is essential. However, the indicator is non-specific for special military missions.

This study aims to describe the perceived well-being among responders deployed in isolated or emergency international missions and provide practical references for intervention development in similar missions.

## Methods

### Design

Compared to quantitative research, qualitative research is more suitable for studying the complexities and subtleties of the world, as it can explore the psychological changes and emotional ups and downs of the participants in an interactive exchange. Research on personnel urgently deployed to perform emergency security or rescue missions in isolated and difficult circumstances is limited.

We conducted in-depth qualitative interviews with participants face to face to gain an understanding of their psychological feelings and emotional experiences and the psychological changes that occur with the prolongation of their missions.

### Participants

The personnel who were urgently deployed to carry out special assignments in isolated and enclosed environments for over a year are included in this study. The inclusion criteria were personnel under closed management for 11 months and willing to participate voluntarily. Those who frequently went out from the campsite for any reason were excluded. We selected the participants by purposive sampling based on the principle of maximum differentiation.

### Materials

The researchers prepared the first draft of the interview outline. Then the research team discussed and revised the interview outline. Five experts of relevant fields reviewed the and approved the interview outline. Before the formal interview, one of the researchers conducted pre-interviews with two participants to test the feasibility and pertinency of the outline. The formal interview outline was finalized based on these pre-interviews. Interview questions about this study focused on the participants’ lived experiences related to habituation, discomfort, perceived burdensomeness, and lack of belonging during the post-deployment transition. Specifically, participants were asked the following questions in a semi-structured interview:

1. What was your immediate thought when informed of the mission notification?
2. What aspects of the mission made you feel stressed? What are the manifestations (cognitive, emotional, mental, physical, behavioral) of that stress? What are the new manifestations that are emerging?
3. How did your psychological status change during the mission, and why?
4. What factors in the mission had the most significant impact on you psychologically?
5. How did you cope with the stress that you felt?
6. What measures have your family and society taken to help you cope with the challenges? How do they work?
7. How would you feel if you were to perform the same mission again?
8. What psychological maintenance advice do you have for people on similar long-term missions in the future?

### Data collection procedures

During the formal interview, the researcher first issued an informed consent form to the interviewees, described the nature and objectives of the study, and clarified the ethical principles and recording request. If the interviewee consented, they signed the informed and recording consent forms. The interviewees had the option to refuse or discontinue the interview. Once consent was obtained, the interviewer assisted the interviewees in filling out a brief form to get the sociodemographic information including gender, age, job, years of working, experience of similar mission, family status, marital status, and whether they have a child. the interview began.

The individual interview was arranged in a bright, quiet room without bystander intervention, which lasted 30 to 40 min. Interview questions were open-ended, and the interviewer was instructed to probe with follow-up questions. Before the interview, the respondents were informed of their right to withdraw consent.

The interviewer was instructed to follow three principles during the interview. First, the interviewer should always remain neutral and may pursue details without asking leading questions. Second, the interviewer must make the interviewees feel understood and accepted instead of arbitrarily judged. Third, the interviewer should use open questions to guide the interviewees to discuss the issues comprehensively. The interviewer used interview strategies such as rhetorical questions, follow-up questions, retelling, and summary, and also recorded facial expressions, body language, and other nonverbal signals.

Data collection continued until data saturation.

### Data analysis

All the sessions were digitally recorded and transcribed separately by two team members. The researcher confirmed the consistency of the two transcribed texts. When discrepancies were found, the original audio was referenced to correct them, and the transcripts were returned to the interviewees for confirmation. Data were analyzed using Colaizzi’s method with the following steps: (1) the researchers familiarized themselves with the data; (2) meaningful statements were identified; (3) recurring ideas were coded, and their meanings were constructed; (4) all results were integrated into themes; (5) original statements were extracted for detailed description; (6) themes were sublimated, and the basic structure was produced; (7) the results were returned to the interviewees to verify the basic structure.

### Ethical considerations

This study was approved by the Committee on Ethics of Medical Research of Naval Medical University (NMUMREC-2021-019). Written informed consent was obtained from all participants. The ‘informed consent’ refers to the participants being aware of the study purpose, risks, and benefits.

## Results

### Sociodemographic characteristics of participants

A total of 11 people participated in the semi-structured interviews, including 10 men and one woman, with ages ranging from 25 to 44 (31.653 ± 5.648) years and an average working time ranging from 5 to 24 (11.727 ± 5.497) years. Among them, three were smokers, seven were married, and five had children. Two (18.18%) of them have worked over 15 years. All were from management, logistics, security, or medical support positions. The information is represented in Table 1.

**Table 1:** The sociodemographic characteristics of participants (*N* = 11)

Item		Category	Number (%)	Item	Category	Number (%)
Gender		Male	10 (90.91%)	Smoking	Yes	3 (27.27%)
		Female	1 (9.09%)		No	8 (72.73%)
Marriage status		Married	7 (63.64%)	Working time	5–10 years	5 (45.46%)
		Single	4 (36.36%)		11–15 years	4 (36.36%)
					> 15 years	2 (18.18%)

Four major themes were identified: ‘perceived somatic change,’ ‘perceived emotional change,’ ‘behavioral change,’ and ‘coping with perceived change.’

### Theme 1 - perceived somatic change

Aid deployment requires a forced change of living environment. Thus, emergency personnel are prone to abnormal immune system function, leading to physical and psychological diseases. Seven participants in the interviews reported physical discomfort, the most common form of sleep disorder. Sleep is essential because it is closely associated with physical and mental health.

I’ve been sleeping so badly in recent months that I often couldn’t fall asleep until 4 am.

Now, the only way I can get a good sleep is to go to the doctor for sleeping pills.

Two participants expressed fatigue, chest tightness, and shortness of breath.

I often experience chest tightness and headaches….

One participant complained about weight loss.

I eat a lot every day, but I’m becoming thinner and thinner. Is there something wrong with my body?

Furthermore, some participants reported skin-related conditions like eczema on their hands, ears, and eyes. These conditions are often closely linked with microenvironment and microecology changes.

I never had eczema before, but I keep getting it since I came here, and it’s not easy to recover.

### Theme 2 - perceived emotional change

The changing working mode and the challenging nature of the work during emergency evacuation could be the leading causes of psychological stress. All the participants said that they felt nervous and anxious.

I had no preparation at all, and it was too sudden.

Maybe I experienced little significant tasks, I was very nervous, and afraid that I might not be qualified.

Furthermore, long-distance separation from relatives during the assistance mission was the main reason for increased psychological burden and emotional changes, manifesting as feelings of guilt and of being lost in their hearts.

I’m separated from my family, unable to accompany my parents, wife, and son, which made me feel very guilty.

I was sent abroad soon after I got married, and I felt a bit lost as I was often out of touch with my wife for such a long time.

My son was just born, but I have not held him even once, so I feel particularly guilty.

In addition, there is minimal external communication due to the isolated and closed nature of the work. The participants reported emotional changes such as low mood and depression.

The monotonous job made me particularly easily fatigued and not really interested in anything. I want something new and exciting.

As the personnel give more time to their mission, their mental state fluctuates. In the middle of the mission, individuals may experience prominent feelings of discomfort, such as irritability, depression, and negativity. They may even exhibit psychological symptoms such as panic, paranoia, and social withdrawal. However, they experience some relief after ending the task.

It was particularly stressful and anxious in the middle of the mission. Now that it’s ending, I feel a bit relieved.

Additionally, significant upheavals such as relationship problems, family difficulties, or major personal physical traumas can induce noticeable fluctuations in psychological and emotional states.

One participant shared, *“Last time when my child had a seizure*,* I was very anxious and helpless. I couldn’t do anything.”*

Another participant mentioned, *“I was initially deployed here for support*,* but due to a work-related injury*,* I had to undergo surgery*,* which added troubles and burdens to our team.”*

### Theme 3 - behavioral change

The emergence and persistent physical and emotional discomfort can induce changes in individuals’ behavior. Five respondents reported experiencing emotional distance and decreased emotional connection frequency.

We are too busy with work. There is noticeably less external communication and interaction.

Four participants mentioned an elevation in their smoking addiction.

I tend to smoke when I’m worried. I used to smoke half a pack a day, but now even a full pack isn’t enough. I think it’s because of missing my family.

Additionally, several participants expressed a lack of interest, a negative work attitude, and a sense of idle time during breaks as negative behavioral changes.

I’m having difficulty focusing on tasks now. I have no interest in anything.

I feel like I’ve lost the motivation and enthusiasm I had when I first arrived.

I just want to sit around and daydream. I can’t get interested in anything.

### Theme 4 - coping with perceived change

#### Theme 4.1 self-adjustment

The interviews revealed that individuals used specific ways to adjust their state of mind, varying from person to person. Primarily, they focused on cultivating personal hobbies and interests and strengthening their persuasion ability, which are effective coping strategies with perceived changes.

I would improve my mood by practicing calligraphy when I am alone.

I’ve found a partner here who also plays guitar, and we often exchange guitar tips in our spare time. Both of us have made great progress.

Some participants also said releasing pressure and negative energy via exercise was their most effective method.

I run each night to release my pressure, then take a shower for a good sleep.

We have to be serious during the usual working hours. However, we can play basketball and volleyball to relax in the evening.

#### Theme 4.2 team support

Interviews revealed that the team organization arranged regular medical follow-ups, psychological screening, and remote psychological treatment to ensure the physical and psychological health of the personnel.

We conduct monthly psychological screening and heart-to-heart chats to keep track of all members’ thoughts in time.

There are general practitioners and nurses on the station to provide support, backed up by remote psychological consultation. All these measures make us very secure.

Most participants reported that proper team support was in place, with accommodation conditions, living support, meals, and campus greenery exceeding their expectations.

We will arrange birthday parties every month and provide barbecue, shabu-shabu, etc.

Besides medical, living, and catering protection, aid team managers established collective activities and various lectures to enrich the emergency personnel’s spare time and control their psychological pressure.

Activities are relatively abundant, such as sports event activities, singing competitions, keynote speeches, group film viewing, and so on.

Various lectures will be arranged every week to enrich everyone’s extra-professional literacy, which is very meaningful.

#### Theme 4.3 social support

Social support consists of solid support for the psychological maintenance of the emergency assistance personnel while relieving their physical and mental burden. The most convenient adjustment path is to improve emotional contact and decrease emotional distance.

We could vent our bad feelings by confiding them.

We can chat with our families and friends, which could relieve a lot of pressure on me.

Besides, family support is a significant factor influencing individuals’ psychological state.

My wife’s support is the key to my perseverance for such a long time.

## Discussion

This study explored the physical and psychological feelings and emotions experienced by emergency assistance personnel during a major public health event and their effective suggestions to cope with perceptual changes while generalizing a program of psychological maintenance strategies. This provides practical experience and timely psychological interventions for the dynamic maintenance of personnel’s physical and psychological health on similar missions.

Under the multiple pressures provided by unfamiliar and isolated environments, stressful tasks, and the inability to take care of their families, emergency assistance personnel are highly susceptible to physical and mental problems. Through qualitative interviews, this study observed that changes in perceived somatic and psychological symptoms were common among the participants. The outcomes indicated that changes in the external environment could significantly affect an individual’s physical and mental health. This includes the occurrence of physical reactions, such as sleep disorders and fatigue; emotional problems, including stress, anxiety, and depression; and behavioral problems, including tobacco addiction and vulnerability to unexpected events. The interviews revealed that sleep disorders and fatigue were the most troubling issues for the personnel. These could endanger personal safety and affect the success or failure of the mission. The fact that some personnel relied upon sleeping pills to fall asleep and were easily awakened is similar to the findings of the research team’s daily consultation in the station [14]. Some of the personnel were found to have physiological and psychological manifestations, including insomnia, easy awakening, alopecia, irritability, and anxiety. These manifestations, represented by severe insomnia, were more pronounced later. Studies outside of China have also shown that it is common for emergency personnel to sleep less than six hours per night [15]. U.S. Army research has validated that more than half of soldiers have sleep problems that have a range of negative effects directly affecting medical readiness [16]. Sleep insufficiency and/or circadian-rhythm disorders may also induce severe mental and physical disorders by deteriorating metabolic, cardiovascular, skeletal muscle, and cognitive health. For instance, poor sleep quality is strongly associated with Alzheimer’s disease in veteran studies [17].

Typically, the mission stress experienced by emergency assistance personnel is considered like that of military mission personnel. Meng-Xue Zhao [18] analyzed the mental health of Chinese plateau military personnel (1993–2017) and found that their psychological health could be easily affected by major military events. Meanwhile, the incidence of psychological problems in individuals during non-war military operations is characterized by a “U” shape: a high incidence of psychological problems during the early stages of the mission, a decline in the mid-mission adaptation stage, and a rise in fatigue stress in the late stage. Therefore, paying close attention to the physical and psychological dynamics of military personnel at different stages of a mission and making targeted improvements are essential.

The study participants proposed specific valuable methods for individuals, units, and society, primarily addressing their material and spiritual needs to alleviate physical and psychological discomfort. To study the implementation of these measures, the research team conducted six symptom checklist-90 scale assessments of the participants and found that their mental health status was better than the norm for military personnel in 2016 [19]. The details are as follows.

Regarding material needs, the conditions of comfortable accommodations and sufficient food and beverages for the personnel can be provided well. First, energy deficiency during work in sustained combat and training operations and other similar, multi-stressor environments has been a longstanding topic of military nutrition research [20]. The importance of focusing on logistics regarding maintaining the mental health of peacekeeping personnel in South Sudan has been mentioned [21]. The study results of the interviews indicated that the scientific development of daily recipes and catering improvement forms, such as the supplemental use of hot pots and barbecues to meet the needs of the personnel, have a positive effect in reducing psychological fluctuations. Moreover, Liu Xue [22] suggested ‘changing the camp environment as much as we can to make the camp alive to maintain the psychological health of officers and soldiers’ via the investigation. The study results also suggest that home-like living conditions can satisfy the personnel’s living needs and alleviate their homesickness.

In terms of spiritual needs, encouraging personnel to develop personal interests and hobbies and enriching cultural, sports, recreational, and leisure activities are also important ways to adjust the mindsets of the personnel. Physical exercise positively affects one’s mental state and alleviates psychological illnesses. A meta-analysis [23] based on 1,331 cases showed that physical exercise significantly reduces depression in adolescents. The study interviews indicate that personal hobbies and the fulfilling use of spare time can reduce stress.

In most cases, social support and active coping were protective anxiety factors [10]. Young and middle-aged men are often subjected to career development and family life. They are the backbone of their families and the mainstay of the workforce, and their mental health problems cannot be ignored [24]. Such individuals can obtain support from professionals (i.e., physicians, psychologists, psychiatrists, employee assistance programs, and chaplains) and non-professional sources (i.e., spouses, friends, colleagues, and leaders) based on participation in mental health training programs and screening for mental health disorders [25].

Finally, we offer three recommendations for the physical and psychological management and maintenance of emergency assistance personnel, considering their feelings and experiences, which is aligned with the coping mechanism of the hardiness model [26]. Hardiness is defined as attitudes that provide the courage and motivation to regard difficult situations as beneficial opportunities and the ability to remain healthy despite stressful circumstances. The general self-efficacy, positive coping, and social support contribute to individual adjustments in face of stress.

First, adequate staff screening and preparation is the first and most critical step before executing a mission. Personnel should be selected with solid professional quality, excellent psychological quality, and high psychological pressure resistance. Before departure, they should complete centralized training so that their psychological readiness will be relatively high. Studies have indicated that military personnel with a high level of psychological preparation tend to have higher efficiency and combat capability [11].

Second, medical staff should regularly conduct psychological assessments, report those with psychological problems, and offer interventions in a timely fashion. However, the mission faced some issues, such as the non-obvious implementation of counseling methods and difficulties coordinating remote expert guidance. Therefore, it is recommended that it should focus on establishing the self-maintenance consciousness of the personnel, encouraging their initiative to achieve active treatment. Psychological lectures should be held regularly to improve the facilitation channels for psychological counseling and intervention. This would also help to reach a timely grasp of sudden and tendentious events about psychological problems. Finally, a relevant feedback mechanism could be established and improved.

Third, physical and psychological adjustment programs must be developed scientifically. This study found that it was common for emergency personnel to have little to do in their spare time. Therefore, we suggest that the development of hobbies be encouraged and that lectures be offered so that each person can realize their value. The social support system should be continuously improved so that the personnel can fully experience a sense of professional value and honorable pride throughout the mission. In addition, many of our participants who had anxiety and insomnia mentioned that they could relieve their distress by running at night. However, recent articles [27] have shown that vigorous exercise before bedtime does not aid sleep but leads to shorter sleep time at night. Therefore, the next step is to explore how to control the amount of exercise, develop a scientific exercise program, and promote physical and mental health.

There are several limitations in this study. Firstly, study results could vary among participants with diverse cultural backgrounds, requiring more generalizability across various cultural contexts. Besides, this study was conducted during a specific mission and may be limited by the responder bias since only 11 participants were involved. However, the sample size of the qualitative research depends on information saturation.

## Conclusions

The mental health status of the emergency personnel was affected by multiple factors in external and internal environments during the emergency mission. This study explored the physical and psychological feelings and emotions experienced by emergency assistance personnel during an emergency mission and their suggestions to adjust mission organization and management. It summarized strategies for maintaining the personnel’s physical and psychological status in an emergency and provided a practical reference for health management in similar missions.

## Data Availability

The data supporting this study’s findings are available from the corresponding author upon reasonable request.

## References

[1] Xia L, Jiang J, Wang J (2017). Comparative study of Chinese and foreign military mental health. China J Health Psychol.

[2] Kang S, Ko J, Kim Y (2012). Development of the stress diagnostic scale on samples of Korean military personnel. Korean J Psychology: Gen.

[3] Wang AX. Research on the construction of early warning indicators for psychological crises in the military. National University of Defense Technology. National University of Defense Technology; 2018.

[4] Lee JE, Choi B, Lee Y, Kim KM, Kim D, Park TW, Lim MH (2023). The relationship between posttraumatic embitterment disorder and stress, Depression, Self-Esteem, Impulsiveness, and suicidal ideation in Korea soldiers in the local area. J Korean Med Sci.

[5] Hacker Hughes J, McCauley M, Wilson L (2019). History of military psychology. J R Army Med Corps.

[6] Moore BA, Barnett JE, Bret A, Barnett, Jeffrey E. Military psychologists’ desk reference. Military psychologists’ desk reference; 2013.

[7] Khorram-Manesh A, Mortelmans LJ, Robinson Y, Burkle FM, Goniewicz K. Civilian-military collaboration before and during COVID-19 Pandemic—A systematic review and a pilot survey among practitioners. Sustainability. 2022;14(2):1–27.

[8] Marvin G, Schram B, Orr R, Canetti EFD. Occupation-induced fatigue and impacts on Emergency First responders: a systematic review. Int J Environ Res Public Health 2023;20(22):7055. 10.3390/ijerph20227055 PMC10671419 37998287

[9] Beckner ME, Lieberman HR, Hatch-McChesney A, Allen JT, Niro PJ, Thompson LA, Karl JP, Gwin JA, Margolis LM, Hennigar SR (2023). Effects of energy balance on cognitive performance, risk-taking, ambulatory vigilance and mood during simulated military sustained operations (SUSOPS). Physiol Behav.

[10] Pankratz L, Sommer JL, Bolton SL, Sareen J, Enns MW, Afifi TO, El-Gabalawy R, Mota N (2022). Prevalence and predictors of anxiety disorder courses in the Canadian Armed forces. J Anxiety Disord.

[11] Feng ZZ, Xia L (2018). Characteristics and influencing factors of psychological problems of Chinese soldiers in non-war military operations. J Third Mil Med Univ.

[12] Toblin RL, Adrian AL, Hoge CW, Adler AB (2018). Energy drink use in U.S. service members after deployment: associations with mental health problems, aggression, and fatigue. Mil Med.

[13] Brooks SK, Greenberg N (2022). Mental health and psychological wellbeing of maritime personnel: a systematic review. BMC Psychol.

[14] Chen Q, Yu M, Peng W, Wei C (2022). Exploring the way of treatment for officers and soldiers in a special geographical environment of a ministry. Health Service J Chin PLA.

[15] Miller NL, Shattuck LG (2005). Sleep patterns of young men and women enrolled at the United States Military Academy: results from year 1 of a 4-year longitudinal study. Sleep.

[16] Devine JK, Collen J, Choynowski JJ, Capaldi V (2020). Sleep disturbances and predictors of nondeployability among active-duty Army soldiers: an odds ratio analysis of medical healthcare data from fiscal year 2018. Mil Med Res.

[17] Raza Z, Hussain SF, Ftouni S, Spitz G, Caplin N, Foster RG, Gomes RSM (2021). Dementia in military and veteran populations: a review of risk factors-traumatic brain injury, post-traumatic stress disorder, deployment, and sleep. Mil Med Res.

[18] Zhao M, Feng Z, Yang G (2019). Improvement of mental health among Chinese plateau military personnel, 1993–2017: a cross-temporal meta-analysis of the Symptom Checklist-90. Neuropsychiatr Dis Treat.

[19] Qiu C, Miao Y, Wang P, Chen W. Analysis of psychological dynamic changes and crowd differences of resident officers and soldiers in isolated and confined environment. *Mil Nurs*.

[20] Karl JP, Margolis LM, Fallowfield JL, Child RB, Martin NM, McClung JP (2022). Military nutrition research: contemporary issues, state of the science and future directions. Eur J Sport Sci.

[21] Liang XM, Mao GF, Tong XL (2019). Practices for maintaining mental health among South Sudanese peacekeepers. People Mil Surg.

[22] Liu X (2019). Investigation of the influencing factors of mental health among military personnel in the plateau area. Manag Observer.

[23] Feng GX, Huang ML. Research progress in status of military psychological stress. World Latest Med Inf. 2019;19(93):49–50,52.

[24] Zhu HT (2020). Impact of mental stress on fatigue and the moderation effect of optimism of military personnel in field training. Mil Med J S Chin.

[25] Andrews KL, Jamshidi L, Nisbet J, Teckchandani TA, Price JAB, Ricciardelli R, Anderson GS, Carleton RN. Mental health training, attitudes toward support, and screening positive for mental disorders among Canadian Coast Guard and Conservation and Protection Officer. Int J Environ Res Public Health. 2022;19(23):15734. 10.3390/ijerph192315734 PMC9739214 36497809

[26] Li XM. Study on the norm development and the mechanism of hardiness among Chinese military personnel. Shanghai: Naval Medical University; 2019.

[27] Pesonen AK, Kahn M, Kuula L, Korhonen T, Leinonen L, Martinmäki K, Gradisar M, Lipsanen J (2022). Sleep and physical activity - the dynamics of bi-directional influences over a fortnight. BMC Public Health.

